# Multiplex CRISPR/Cas9-based genome engineering from a single lentiviral vector

**DOI:** 10.1093/nar/gku749

**Published:** 2014-08-13

**Authors:** Ami M. Kabadi, David G. Ousterout, Isaac B. Hilton, Charles A. Gersbach

**Affiliations:** 1 Department of Biomedical Engineering, Duke University, Durham, NC 27708, USA; 2 Center for Genomic and Computational Biology, Duke University, Durham, NC 27708, USA; 3 Department of Orthopaedic Surgery, Duke University Medical Center, Durham, NC 27710, USA

## Abstract

Engineered DNA-binding proteins that manipulate the human genome and transcriptome have enabled rapid advances in biomedical research. In particular, the RNA-guided CRISPR/Cas9 system has recently been engineered to create site-specific double-strand breaks for genome editing or to direct targeted transcriptional regulation. A unique capability of the CRISPR/Cas9 system is multiplex genome engineering by delivering a single Cas9 enzyme and two or more single guide RNAs (sgRNAs) targeted to distinct genomic sites. This approach can be used to simultaneously create multiple DNA breaks or to target multiple transcriptional activators to a single promoter for synergistic enhancement of gene induction. To address the need for uniform and sustained delivery of multiplex CRISPR/Cas9-based genome engineering tools, we developed a single lentiviral system to express a Cas9 variant, a reporter gene and up to four sgRNAs from independent RNA polymerase III promoters that are incorporated into the vector by a convenient Golden Gate cloning method. Each sgRNA is efficiently expressed and can mediate multiplex gene editing and sustained transcriptional activation in immortalized and primary human cells. This delivery system will be significant to enabling the potential of CRISPR/Cas9-based multiplex genome engineering in diverse cell types.

## INTRODUCTION

Recent discoveries of novel protein–DNA interactions in various species and systems have led to the development of methods for engineering synthetic proteins that can be targeted to predefined DNA sequences (1–4). These proteins can be fused to diverse effector domains to create new enzymes that modify DNA sequence or transcriptional regulation at any site in the genome. Engineered DNA-binding proteins that manipulate the human genome and transcriptome have enabled important advances in biomedical research, including highly efficient site-specific gene modification (5–12), transcriptional activation (13–20) and epigenetic modification (21–24). Notably, a Phase I clinical study based on *ex vivo* gene editing using zinc-finger nucleases (ZFNs) recently met defined safety criteria (6,12), demonstrating the clinical applicability of site-specific DNA modification technologies.

A significant recent advance in genome engineering is the development of the CRISPR/Cas9 system for nuclease-based genome editing (25–29) and also for transcriptional regulation (15,18,20,22,29–35). In this system, a chimeric single guide RNA (sgRNA) is utilized to direct the Cas9 protein to predefined DNA sequences (36). The sgRNA can be targeted to any desired DNA sequence by exchanging the 20 bp protospacer that confers targeting specificity through complementary base pairing with the desired DNA target. The natural function of Cas9 is to act as a nuclease by creating DNA double-stranded breaks via RuvC and HNH endonuclease domains, each of which cleaves one strand of the target DNA (37). The nuclease domains can be inactivated by two amino acid substitutions (D10A and H840A) to generate a Cas9 protein that has no endonuclease activity but maintains its RNA-guided DNA-binding capacity (36). This deactivated Cas9 (dCas9), in conjunction with the sgRNA, functions as a modular DNA-binding scaffold. We and others have previously shown that this DNA-binding scaffold can be used to mediate gene regulation by fusion of the VP64 transactivation domain or KRAB repression domain to dCas9 (15,18,20,22,29–35). Unlike other genome engineering systems based on zinc finger proteins and transcription activator-like effectors (1,2,4), novel proteins do not need to be engineered for each CRISPR/Cas9 target sequence. Therefore, this technology greatly expedites the process of molecular targeting to new sites by simply modifying the expressed sgRNA molecule.

The CRISPR/Cas9 system can simultaneously target multiple distinct genomic loci by co-expressing a single Cas9 protein with two or more sgRNAs, making this system uniquely suited for multiplex gene editing (25,26) or synergistic activation of target genes (15,18). In most cases, this has been achieved by encoding the Cas9 variant and each sgRNA on independent plasmids that are mixed for co-transfection. However, co-transfection of multiple plasmids leads to variable expression levels in each cell due to differences in copy number. Additionally, gene activation following transfection is transient due to dilution of plasmid DNA over time. Moreover, many cell types are not easily transfected and transient gene expression may not be sufficient for achieving the desired effect. To address these limitations, we have developed a user-friendly platform to express Cas9 or a dCas9 variant, a reporter gene and up to four sgRNAs, each from independent Pol III promoters, from a single lentiviral vector. The final vector is readily generated by Golden Gate assembly. We show that each promoter is capable of efficiently expressing sgRNAs that direct similar levels of Cas9 nuclease activity. Furthermore, lentiviral delivery of a single vector expressing Cas9 and four sgRNAs targeting distinct loci resulted in simultaneous multiplex gene editing of all four loci. Finally, we demonstrate tunable transcriptional activation at two endogenous genes that can be either transient or sustained using lentiviral delivery of dCas9^VP64^ with or without sgRNAs. Notably, we observed efficient and long-term targeted activation of endogenous genes in primary human cells, highlighting the utility and power of this approach. This system is therefore an attractive and efficient method to facilitate multiplex gene editing and long-term transcriptional activation in human cells.

## MATERIALS AND METHODS

### Plasmid constructs

The gene encoding human codon optimized Cas9 (hCas9) nuclease was obtained from Addgene (Plasmid #41815) (25), and we reported the expression cassette for the *Streptococcus pyogenes* sgRNA previously (Addgene Plasmid #47108) (18). Additional promoters for mU6 (38), H1 (39) and 7SK (40) Pol III promoters were synthesized using GeneBlocks (IDT) and cloned in place of the hU6 sgRNA expression cassette. A GeneBlock (IDT) was inserted into the 3′ of the hCas9 coding sequence to fuse a T2A skipping peptide and eGFP gene to monitor Cas9 expression. The coding sequence for hCas9-T2A-GFP was then transferred into the FUGW lentiviral expression vector (Addgene Plasmid #14883) containing the human ubiquitin C (hUbC) promoter to drive expression of hCas9-T2A-GFP, as well as restriction sites to facilitate Golden Gate cloning of sgRNA expression cassettes immediately upstream of the hUbC promoter (Figure 2). All plasmids have been made publicly available through the Addgene non-profit plasmid repository (plasmids #53186–53192).

**Figure 1.**
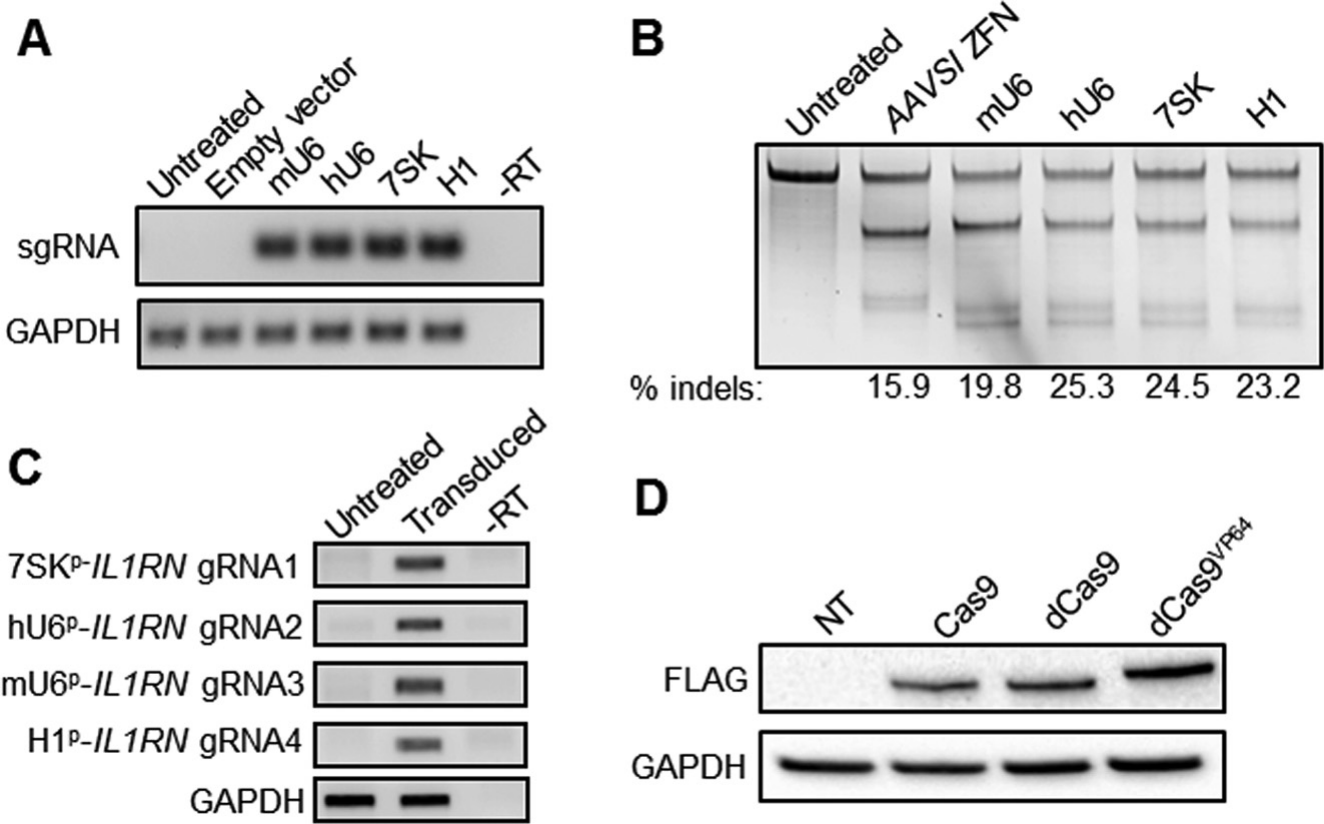
Validation of sgRNA and lentiviral Cas9 expression constructs. (**A**) Constructs encoding unique Pol III promoters expressing sgRNAs targeting the AAVS1 locus or a construct containing the hU6 promoter immediately followed by poly-thymidine to terminate expression (‘Empty vector’) were transfected into HEK293T cells. End-point RT-PCR was used to probe for expression of each indicated promoter/sgRNA construct 2 days post-transfection. –RT: no reverse transcription control. (**B**) HEK293T cells were transfected with expression vectors encoding the *AAVS1*-targeted zinc finger nuclease or Cas9-T2A-GFP and the indicated promoter/sgRNA expression cassettes and assessed for gene modification levels 3 days post-transfection using the Surveyor assay. (**C**) HEK293T cells were transduced with a single lentiviral vector expressing four sgRNAs targeting the *IL1RN* promoter. End-point RT-PCR was used to probe for expression of each indicated promoter/sgRNA construct at 10 days post-transduction. –RT: no reverse transcription control. (**D**) HEK293T cells were transduced with lentiviral constructs encoding the indicated Cas9-T2A-GFP constructs without sgRNAs and assessed for Cas9 expression by western blot 7 days post-transduction by probing for a FLAG epitope tag on the N-terminus of the Cas9 protein.

**Figure 2.**
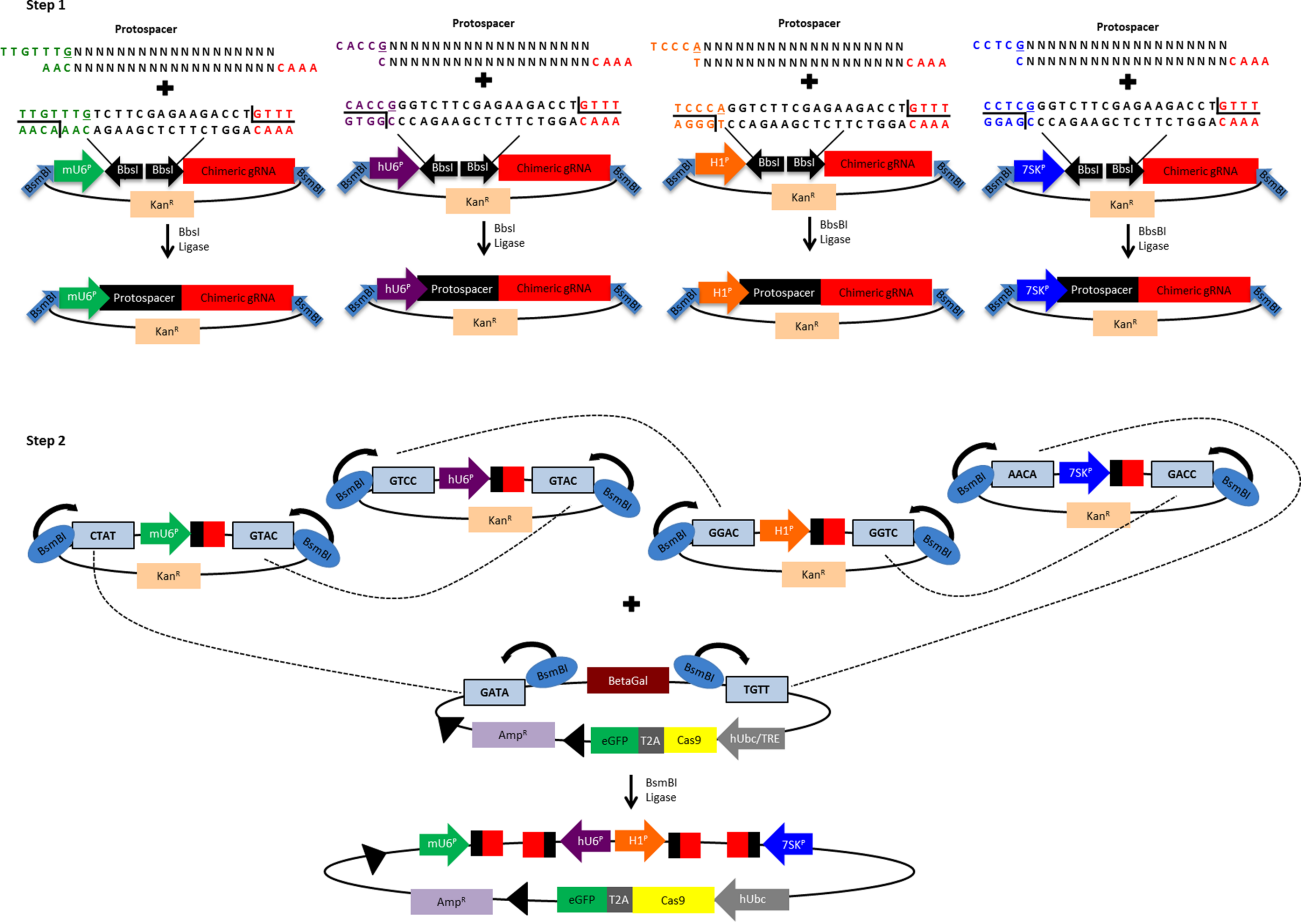
Golden Gate assembly of single lentiviral vectors encoding CRISPR/Cas9 and multiple sgRNA expression cassettes. In the first step, individual sgRNAs are cloned into separate expression vectors and sequence verified. Fragments containing each sgRNA expression cassette are then transferred into a Cas9-expressing lentivirus in step 2 by Golden Gate assembly as shown. Black triangles shown in the lentiviral plasmid represent *loxP* sites flanking the entire sgRNA and Cas9 expression cassette.

### Protocol for assembly of custom lentiviral vectors

Assembly of custom lentiviral vectors expressing up to four sgRNAs of choice and active Cas9, dCas9 or dCas9^VP64^ can be accomplished in <5 days. The cloning method makes use of Golden Gate cloning and type IIS restriction enzymes that cleave outside their recognition sequence to create unique overhangs. Golden Gate assembly expedites cloning as all four expression cassettes are ligated into the final lentiviral vector in one step. The lentiviral vector expresses either active Cas9, dCas9 or dCas9^VP64^ in addition to one, two, three or four sgRNAs expressed from independent promoters.

*Step 1: Anneal single stranded oligos containing each 20 bp protospacer to create overhangs for ligation into the desired donor promoter vector*. Two single-stranded oligos for each desired genomic target can be designed using the template provided in Supplementary Table S1. To anneal the complimentary oligos, 8 μl sense oligo + 8 μl antisense oligo (both at 10 μM) + 2 μl 10× ligase buffer (NEB) are mixed, followed by melting and reannealing in a thermal cycler with the program: 96°C for 300 s, followed by 85°C for 20 s, 75°C for 20 s, 65°C for 20 s, 55°C for 20 s, 45°C for 20 s, 35°C for 20 s and 25°C for 20 s with a −0.3°C /s rate between steps. To phosphorylate the overhangs, 1 μl 25 mM ATP + 1 μl T4 Polynucleotide Kinase (NEB) are added and incubated at 37°C for 60 min followed by 65°C for 20 min to heat inactivate the enzyme. Each protospacer was ligated into the desired expression vector using T4 DNA ligase (NEB) incubated at 16°C for 60 min using 50 ng of vector and 1 μl of annealed oligonucleotides in a 10 μl reaction volume according to manufacturer's instructions. Five microliters of each ligation was transformed into XL1 blue chemically competent bacteria (Agilent) following the manufacturer's instructions. Transformations were plated onto LB agar plates containing 50 μg/ml kanamycin (Sigma) and incubated overnight at 37°C. Typically, >90% of the colonies contain the desired ligation product. Sequencing can be performed using the M13 reverse standard sequencing primer to validate each final sgRNA construct prior to proceeding to step 2.
*Step 2: Transfer the four promoter-gRNA cassettes into a lentiviral destination vector using Golden Gate assembly*. After completion of step 1, there are four independent plasmids each expressing a different sgRNA from a different promoter. To assemble the four different promoter-sgRNA constructs into the desired destination vector, 200 ng of each sgRNA expression plasmid and the desired lentiviral destination vector are combined with 1 μl of T4 DNA ligase (NEB), 1 μl BsmBI FastDigest (Fisher Scientific) and 2 μl 10× T4 ligase buffer (NEB) in a 20 μl reaction volume. The reaction is incubated as follows: 37°C for 10 min, 16°C for 15 min, 37°C for 30 min, 80°C for 5 min. Five microliter of ligation reaction is transformed into SURE 2 chemically competent cells (Agilent) following the manufacturer's instructions. Transformations are plated onto LB agar plates containing 100 μg/ml ampicillin and incubated overnight at 37°C. Optionally, colonies can be screened by lacZ-based blue/white screening using IPTG and X-gal; however, in our experience, this is often unnecessary as >90% of the transformants contain the proper ligation product. Due to the inverted repeats formed by the opposing sgRNA expression cassettes, the final constructs may be unstable and thus we recommend maintaining these plasmids in the SURE 2 cell line and screening the final plasmid with a test PCR using the sense primer 5′-TCGGGTTTATTACAGGGACAGCAG-3′ and antisense primer 5′-TCTAAGGCCGAGTCTTATGAGCAG-3′. These primers amplify across the four promoter-gRNA region. Due to the repetitive nature, a distinct banding pattern should be observed as shown in Supplementary Figure S1 with the largest product ∼1800 bp in size.

### Cell culture and transfection

HEK293T cells were obtained from the American Tissue Collection Center (ATCC, Manassas, VA, USA) through the Duke University Cancer Center Facilities and were maintained in Dulbecco's modified Eagle's medium (DMEM) supplemented with 10% FBS and 1% penicillin/streptomycin. Primary human dermal fibroblasts (Catalog ID: GM03348) were obtained from Coriell Institute (Camden, NJ, USA) and were maintained in DMEM supplemented with 10% FBS and 1% penicillin/streptomycin. All cells were cultured at 37°C with 5% CO_2_. HEK293T cells were transfected with Lipofectamine 2000 (Life Technologies) with 200 ng of each sgRNA expression vector (800 ng total pDNA) according to the manufacturer's protocol in 24 well plates. For derivation of clonal populations from single cells, HEK293T cells were transduced with lentivirus expressing active Cas9 and 4 sgRNA cassettes and grown in culture for 7 days. After 7 days, single cell clones were isolated by limiting dilution in 96 well plates. Clones expressing GFP were expanded and genomic DNA was harvested for analysis.

### Viral production and transduction

All lentiviral vectors used is this study are second generation and were produced using standard viral production methods that have been previously described (41). Briefly, 3.5 million HEK293T cells were plated per 10 cm dish. The following day, cells were transfected by the calcium phosphate transfection method with 20 μg of transfer vector, 6 μg of pMD2G and 10 μg psPAX2. The media was changed 12–14 h post-transfection. The viral supernatant was collected 24 and 48 h after this media change, passed through a 0.45 μm filter, pooled and used either fresh or snap frozen. For transduction, cells were resuspended and plated in viral supernatant supplemented with 4 μg/ml polybrene. The viral supernatant was exchanged for fresh medium 12–24 h later.

A lentivirus titer protocol was adapted from methods previously described (41). Briefly, 2.5 × 10^4^ HEK293T cells were plated in serial dilutions of virus in the presence of 4 μg/ml polybrene. One day later, the viral supernatant was replaced with fresh media. Cells were analyzed for the percentage of GFP positive cells using a BD FACSCalibur 4 days post-transduction. Titer was calculated as follows:

\begin{eqnarray*} &&{\rm Titer}\left( {\frac{{{\rm HEK293T\;transducing\;units}}}{{{\rm ml\;virus}}}} \right) = \nonumber \\ &&\frac{{2.5 \times 10^4 ({\rm target\;HEK293T\;cells}) \times \frac{{\% {\rm GFP\;positive\;cells}}}{{100}}}}{{{\rm volume\;viral\;supernatant} ({\rm ml})}} \end{eqnarray*}

Because cells were plated directly into virus, the starting cell number was used, in contrast to the published protocol that transduces the following day and estimates a corresponding doubling in cell number (41).

### Reverse transcription-PCR

RNA was isolated using the miRNeasy Mini RNA isolation kit (Qiagen). DNAse digestion was performed using the DNA-free Kit (Applied Biosystems). cDNA synthesis was performed using the SuperScript VILO cDNA Synthesis Kit (Invitrogen). cDNA was amplified by PCR using Taq DNA polymerase (NEB) and the resulting product was run on TAE agarose gels. Oligonucleotide primers and PCR conditions are reported in Supplementary Table S2. Images were captured using a ChemiDoc XRS+ System and processed using ImageLab software (Bio-Rad).

### Quantitative reverse transcription-PCR

RNA was isolated using the RNeasy Plus RNA isolation kit (Qiagen). cDNA synthesis was performed using the SuperScript VILO cDNA Synthesis Kit (Invitrogen). Real-time PCR using PerfeCTa SYBR Green FastMix (Quanta Biosciences) was performed with the CFX96 Real-Time PCR Detection System (Bio-Rad). Oligonucleotide primers and PCR conditions are reported in Supplementary Table S2. Primer specificity was confirmed by agarose gel electrophoresis and melting curve analysis. Reaction efficiencies over the appropriate dynamic range were calculated to ensure linearity of the standard curve (Supplementary Figure S2). The results are expressed as fold-increase expression of the gene of interest normalized to *GAPDH* expression using the ΔΔC_t_ method. Reported values are the mean and standard error of the mean from two independent experiments (*n* = 4).

### Western blot

Cells were lysed in RIPA Buffer (Sigma) supplemented with protease inhibitor cocktail (Sigma). Protein concentration was measured using BCA protein assay reagent (Thermo Scientific) and BioTek Synergy 2 Multi-Mode Microplate Reader. Lysates were mixed with loading buffer and boiled for 5 min; twenty-five micrograms of protein were run in NuPage 4–12% Bis-Tris Gel polyacrylamide gels (Bio-Rad) and transferred to nitrocellulose membranes. Nonspecific antibody binding was blocked with TBS-T (50 mM Tris, 150 mM NaCl and 0.1% Tween-20) with 5% nonfat milk for 1 h at room temperature. The membranes were incubated with primary antibodies: anti-FLAG-HRP (1:1000, Cell Signaling 2044) in 5% milk in TBS-T for 60 min at room temperature; anti-GAPDH (1:5000, Cell Signaling, clone 14C10) in 5% milk in TBS-T for 30 min at room temperature. Membranes were then washed three times with TBS-T for 15 min total. Membranes were incubated with anti-rabbit horseradish peroxidase (HRP)-conjugated antibody (Sigma, A 6154) or anti-mouse HRP-conjugated antibody (Santa Cruz, SC-2005) diluted 1:5000 for 30 min and washed with TBS-T three times for 15 min each. Membranes were visualized using the ImmunStar WesternC Chemiluminescence Kit (Bio-Rad) and images were captured using a ChemiDoc XRS+ System and processed using ImageLab software (Bio-Rad).

### Cel-I quantification of endogenous gene modification

CRISPR/Cas9 nuclease lesions at the endogenous target site were quantified using the Surveyor nuclease assay, which can detect mutations characteristic of nuclease-induced DNA repair by non-homologous end joining (42). After transfection or transduction, cells were incubated for 3–10 days at 37°C and genomic DNA was extracted using the DNeasy Blood and Tissue kit (Qiagen). The target locus was amplified by 35 cycles of PCR with the AccuPrime High Fidelity PCR kit (Invitrogen) using primers indicated in Supplementary Table S2. The resulting PCR products were randomly melted and reannealed in a PCR machine with the program: 95°C for 240 s, followed by 85°C for 60 s, 75°C for 60 s, 65°C for 60 s, 55°C for 60 s, 45°C for 60 s, 35°C for 60 s and 25°C for 60 s with a −0.3°C /s rate between steps. Following reannealing, 8 μl of PCR product was mixed with 1 μl of Surveyor Nuclease S and 1 μl of Enhancer S (Transgenomic, Omaha, NE, USA) and incubated at 42°C for 1 h. After incubation, 6 μl of digestion product was loaded onto a 10% TBE polyacrylamide gel and run at 200 V for 30 min. The gels were stained with ethidium bromide and quantified using ImageLab (Bio-Rad) by densitometry as previously described (43).

### Statistical analysis

At least two independent experiments each consisting of two independent transfections or transductions were compiled as means and standard errors of the mean (*n* = 4). Effects were evaluated with multivariate ANOVA and Dunnett's post hoc test using JMP 10 Pro.

## RESULTS

### Development of a single lentiviral vector for multiplex genome engineering

A current challenge for implementing multiplex CRISPR/Cas9-based genome engineering systems is the simultaneous and efficient delivery of multiple sgRNAs and the Cas9 gene, especially in cell types that are difficult to transfect. To address this challenge, we developed a single lentiviral vector that efficiently expresses Cas9 and up to four sgRNAs, including an eGFP reporter gene co-expressed with Cas9 to facilitate quantification of transduction and/or fluorescence-based sorting of transduced cells (44). In order to minimize potential recombination due to repetitive DNA sequences and to maximize the expression efficiency of each sgRNA, this vector expresses four sgRNAs from four independent Pol III promoters (human U6 promoter, mouse U6 promoter, 7SK and H1) (45). We first validated sgRNA expression from each promoter on independent plasmids using end-point RT-PCR to detect an sgRNA targeting the *AAVS1* locus (Figure 1A). To test the activity of each sgRNA expression construct, we co-transfected a plasmid containing each promoter construct expressing an sgRNA targeting *AAVS1* together with a plasmid containing an active Cas9 expression construct into human HEK293T cells. Notably, we detected consistent and high levels of gene modification at the target locus for each sgRNA that are comparable to a well-characterized ZFN with high activity at the *AAVS1* locus (46,47) (Figure 1B).

We developed a Golden Gate cloning method to facilitate rapid and efficient cloning of multiple sgRNA expression cassettes into a single lentiviral vector expressing the desired Cas9 effector (Figure 2). In the first step, oligonucleotides encoding sgRNA protospacer sequences are cloned independently into different expression vectors with type IIS restriction enzymes (26), each with a distinct promoter driving sgRNA expression. In the second step, each sgRNA expression construct is subcloned by Golden Gate assembly into a lentiviral expression vector encoding a Cas9 or dCas9 variant. This strategy allows for robust and rapid cloning of up to four sgRNAs into a single lentiviral vector for gene editing or activation applications. To express less than four sgRNAs, an empty expression cassette consisting of a poly-T terminator sequence cloned downstream of unused promoters is used to prevent unwanted transcription. Each vector co-expresses the selected Cas9 or dCas9 variant with eGFP via a 2A skipping peptide to enable fluorescence-activated flow sorting and selection of cells with a high multiplicity of infection that can be useful for enriching cells with high levels of gene editing events (44). Finally, the entire region containing the sgRNA and Cas9 expression cassettes is flanked by loxP sites to mediate removal by Cre-lox excision (Supplementary Figure S3). This all-in-one lentiviral system can be effectively packaged into lentiviral particles with titers of ∼6 × 10^4^ transducing units per ml (Supplementary Figure S4). Expression from the lentiviral vector was persistent for several weeks (Supplementary Figure S5).

To validate the expression of each sgRNA from the lentiviral vector, we assembled a single lentiviral vector expressing four independent *IL1RN*-targeting sgRNAs (18) from the four independent Pol III promoters. Using end-point RT-PCR to detect each sgRNA, we observed expression of each sgRNA within the bulk population of transduced cells (Figure 1C). Furthermore, lentiviral delivery of a construct expressing different Cas9 transgenes, including an active Cas9 nuclease, dCas9 or dCas9 fused to the VP64 transactivator domain, resulted in expression of full-length Cas9 protein in HEK293T cells as determined by western blot (Figure 1D).

### Validation of a single lentiviral sgRNA/Cas9 expression vector for multiplex genome engineering

**Figure 3.**
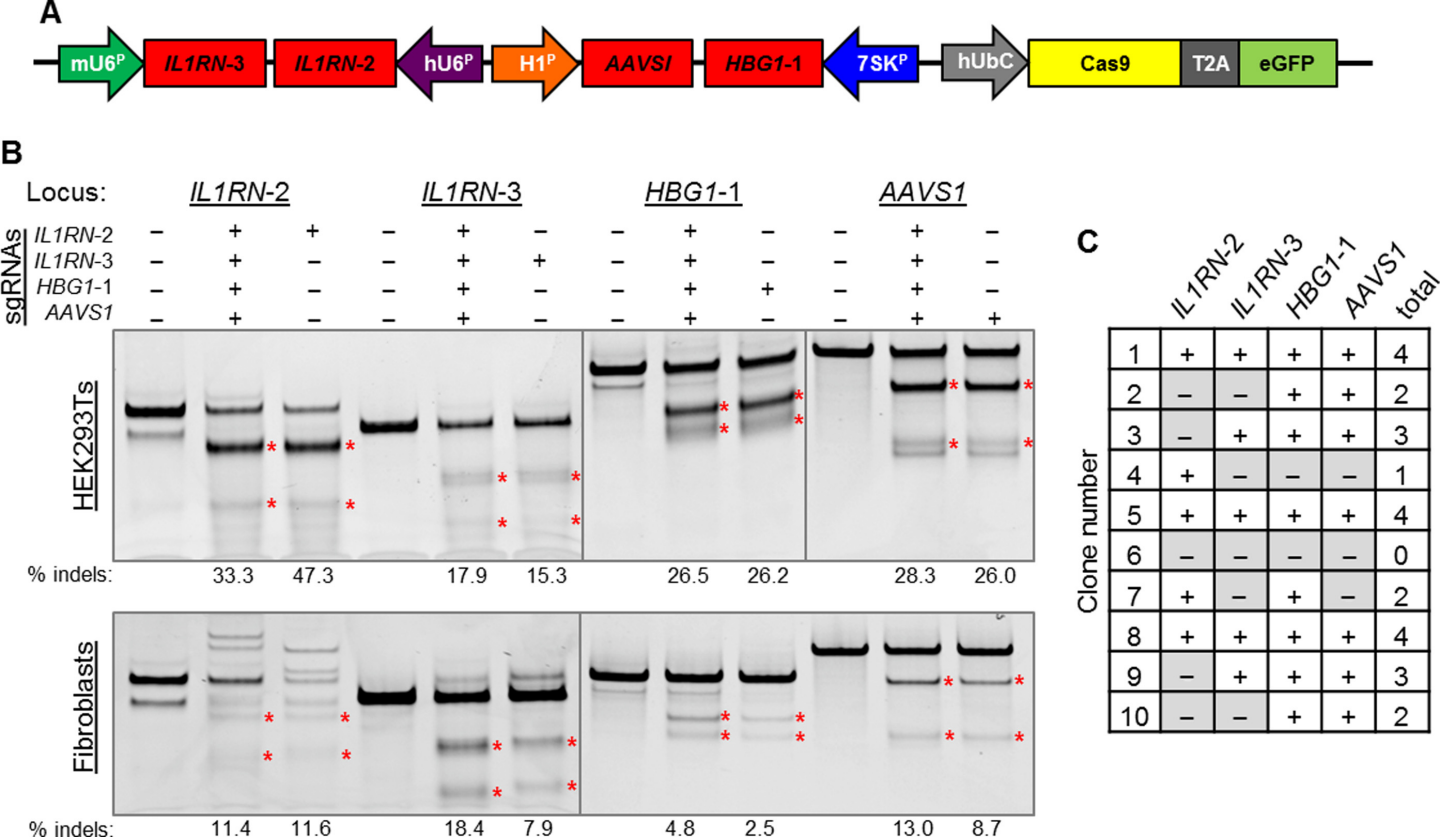
Delivery of a multiplex CRISPR/Cas9 system with a single lentivirus. (**A**) Four sgRNAs targeting distinct genomic loci were cloned into a lentiviral vector expressing the active Cas9 nuclease. (**B**) HEK293T cells and primary human dermal fibroblasts were transduced with lentivirus expressing the indicated sgRNAs and assayed for nuclease activity using the Surveyor assay. HEK293T cells were assayed 7 days post-transduction. The human fibroblasts were assayed 10 days post-transduction. (**C**) Summary of gene editing events at four targeted loci in 10 single cell-derived clonal populations of HEK293T cells transduced with a single lentiviral Cas9 vector. Surveyor analysis for each individual clone is shown in Supplementary Figure S8.

To validate the independent activity of each sgRNA, we assembled a single lentiviral vector expressing active Cas9 and four sgRNAs, each targeting independent loci (Figure 3A). As control vectors, we assembled constructs expressing only one sgRNA along with empty sgRNA expression cassettes in the other three positions. We transduced HEK293Ts and primary fibroblasts with lentiviral vectors expressing the indicated sgRNAs and monitored gene modification frequencies at 7 or 10 days post-transduction, respectively (Figure 3B). In both cell types, the single lentiviral vector mediated highly efficient multiplex editing at all four loci (Figure 3B). Interestingly, expression of all four sgRNAs together resulted in higher modification frequencies than a single sgRNA alone at three out of four loci in fibroblasts (Figure 3B). We observed efficient multiplex gene editing in transduced fibroblasts, which are typically difficult to transfect. Notably, lentiviral transduction of HEK293Ts rapidly introduces gene modifications that are stable for several weeks in culture even in the presence of constitutive Cas9 nuclease expression (Supplementary Figures S5 and S6A–B). A potential concern with this lentiviral system is that the multiple copies of the sgRNA cassettes may spontaneously recombine due to the repetitive sequences in the 3′ end of the sgRNA. To determine the stability of the sgRNA expression cassettes in the integrated viral vector, PCR of the genomic DNA was performed at each time point. PCR bands consistent with full-length sgRNA cassette transfer were detected in the bulk population of transduced HEK293T cells and primary human fibroblasts for up to three weeks (Supplementary Figures S1, S6C and S7). In order to conclusively demonstrate that multiplex gene editing was occurring in single cells, clonal populations were derived from the polyclonal cell population transduced with the construct carrying four sgRNAs (Figure 3A). Ten clonal populations were assessed by the Surveyor assay for gene modification, of which three showed the expected gene editing events at all four loci (Figure 3C and Supplementary Figure S8). Notably, eight of the clones showed editing at two or more loci, indicating that the bulk Surveyor results (Supplementary Figure S6A–B) may be underrepresenting overall editing frequencies after prolonged culture. These data demonstrate that a single lentivirus can express four active sgRNAs efficiently and that this lentiviral platform can be used to target four distinct loci for multiplex CRISPR/Cas9 gene editing.

### Transient RNA-guided gene activation in cell lines stably expressing a lentiviral Cas9-based transactivator

**Figure 4.**
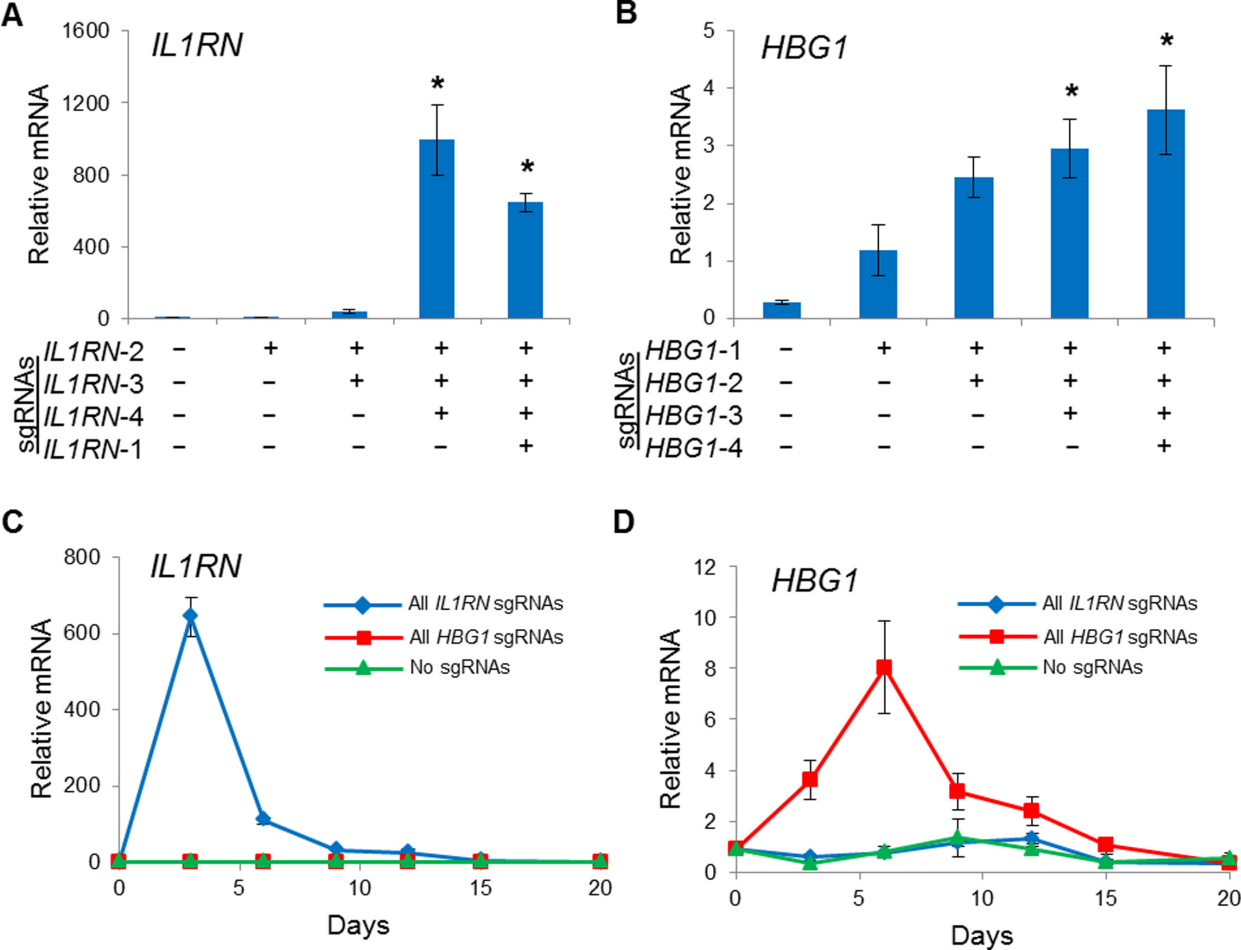
Transient gene activation in HEK293T cells stably expressing dCas9^VP64^. HEK293T cells were transduced with lentivirus to stably express dCas9^VP64^ and were subsequently transfected with plasmid expressing the indicated sgRNA combinations. By varying the number of sgRNAs delivered, the level of endogenous gene activation was tunable for both the (**A**) *IL1RN* and (**B**) *HBG1* loci at 3 days post-transfection (*P < 0.05 versus no sgRNAs). Peak levels of (**C**) *IL1RN* and (**D**) *HBG1* were observed 3–6 days post-transfection and the level of activation returned to background levels between days 15 and 20.

Next, we characterized this system for transient gene activation by transfecting sgRNAs into model cell lines stably expressing dCas9^VP64^. HEK293Ts were transduced with different Cas9-T2A-eGFP constructs and eGFP expression was monitored using flow cytometry. Following normal passaging every 2–3 days, each cell line exhibited stable eGFP expression for up to 35 days post-transduction (Supplementary Figure S5). Transduced HEK293T cells were then co-transfected with one to four separate sgRNA expression constructs on independent plasmids targeting either the *IL1RN* or *HBG1* promoter. Transient transfection of these sgRNA constructs into cells lines stably expressing dCas9^VP64^ resulted in tunable endogenous gene activation (Figure 4A and B), consistent with previous studies of synergistic RNA-guided gene activation (15–18). As expected, gene activation following transient transfection of sgRNA constructs in cells expressing dCas9^VP64^ reached a maximum level of activation ∼3–6 days post-transfection and fell to undetectable levels by 20 days post-transfection (Figure 4C and D). Gene activation was specific to each sgRNA target (Supplementary Figure S9) (18). These data demonstrate that lentiviral dCas9^VP64^ combined with transient sgRNA delivery can be used as a versatile system to tunably and transiently activate endogenous target genes

### Stable gene activation in HEK293T cells using a single lentiviral expression vector

**Figure 5.**
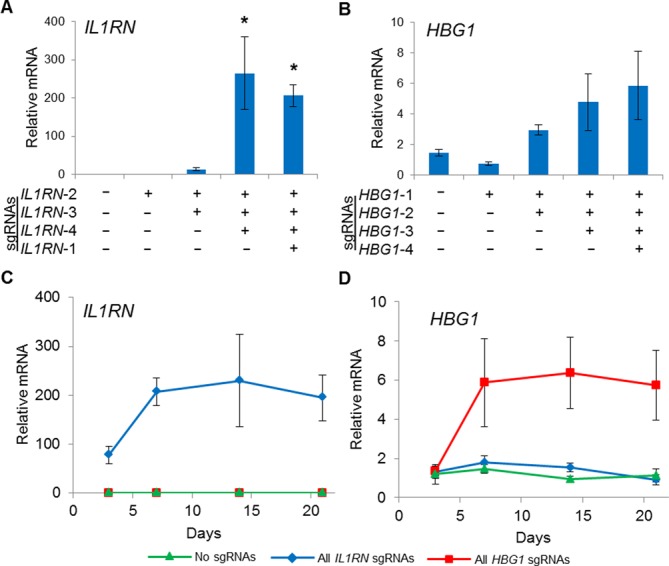
Stable gene activation by dCas9^VP64^ in HEK293T cells using a single multiplex lentiviral vector. HEK293T cells were transduced with lentivirus to stably express dCas9^VP64^ and the indicated combinations of sgRNAs. Activation of the endogenous (**A**) *IL1RN* and (**B**) *HBG1* loci at 7 days post-transduction were tunable by varying the number of sgRNAs delivered (*P < 0.05 versus no sgRNAs). Peak levels of endogenous (**C**) *IL1RN* and (**D**) *HBG1* were observed 6 days post-transduction and the level of activation was sustained through day 21.

Lentiviral delivery may enable stable, long-term gene activation by CRISPR/Cas9 transactivation. To test this, HEK293Ts were transduced using a single lentiviral vector encoding dCas9^VP64^ and one to four sgRNA expression cassettes. Similar to the transient transfection results (Figure 4), we were able to tunably and robustly activate expression of endogenous *IL1RN* and *HBG1* genes (Figure 5A and B). Gene activation induced by co-transfection of HEK293T cells with dCas9^VP64^ and four sgRNAs targeted to the *IL1RN* and *HBG1* promoters peaked between three and five days post-transfection and gene expression returned to background levels 15–20 days post-transfection (Figure 4C and D). In contrast, lentiviral delivery of dCas9^VP64^ and the same four *IL1RN* or *HBG1*-targeted sgRNAs induced sustained gene activation for more than 20 days post-transduction (Figure 5C and D). Thus, single lentiviral delivery of multiplex dCas9^VP64^ transactivators is a useful platform to efficiently and stably upregulate endogenous target genes.

### Stable gene activation in primary human dermal fibroblasts

**Figure 6.**
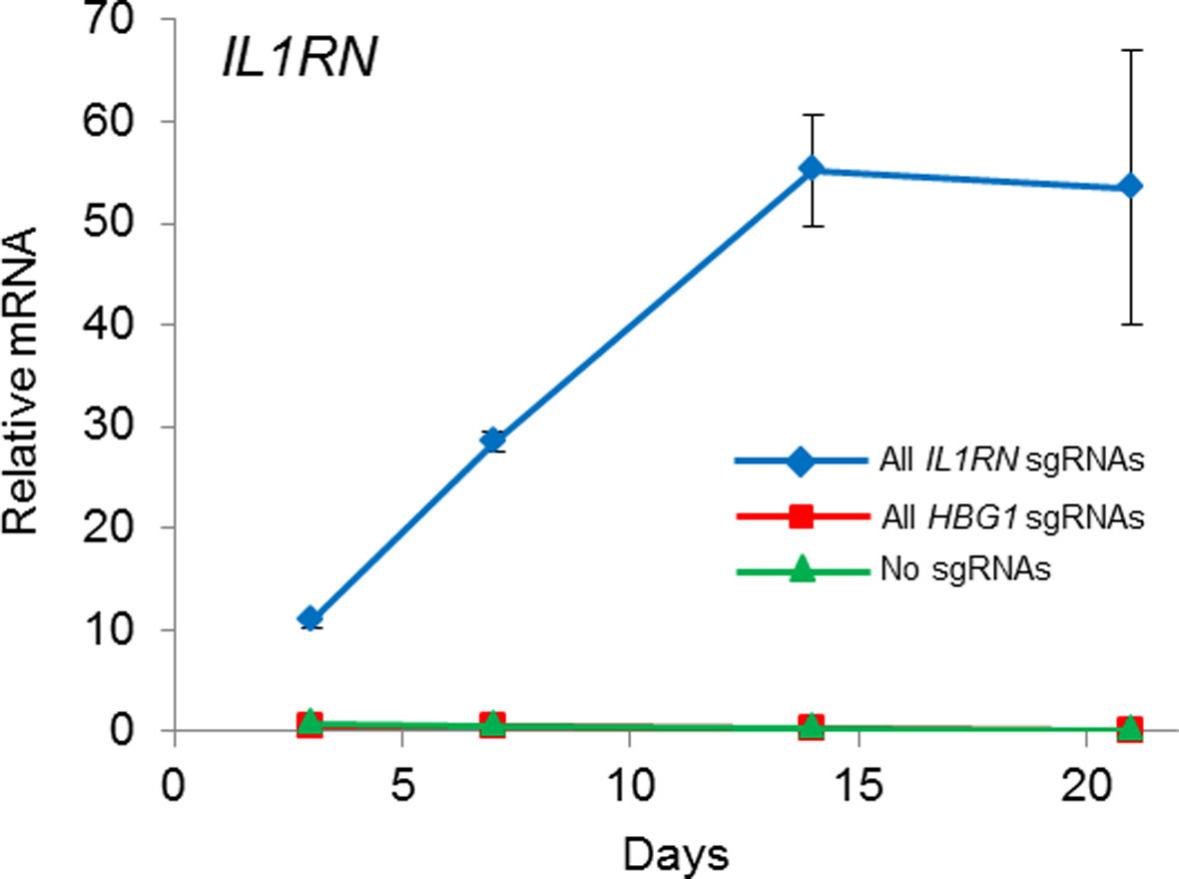
Stable gene activation by dCas9^VP64^ in primary human fibroblasts using a single multiplex lentiviral vector. Primary human dermal fibroblasts were transduced with lentivirus to stably co-express dCas9^VP64^ and four sgRNAs targeted to the *IL1RN* promoter. Peak levels of endogenous *IL1RN* were observed 14 days post-transduction and the level of activation was sustained through day 21.

Many applications require long-term stable gene expression in primary cells that are difficult to transfect. To test the lentiviral vector in this context, we transduced primary human dermal fibroblasts with a single lentivirus co-expressing dCas9^VP64^ and four sgRNAs targeted against the *IL1RN* or *HBG1* endogenous promoters. We observed sustained activation of the *IL1RN* locus for up to 21 days post-transduction (Figure 6). However, we did not observe any activation of the *HBG1* locus (data not shown). This may be because the *HBG1* sgRNAs induce lower levels of gene activation compared to the *IL1RN* sgRNAs (Figure 5C and D). Furthermore, activation in the primary dermal fibroblasts was notably lower than in HEK293T cells (Figures 5C and 6), potentially due to lower overall expression levels or epigenetic context. While our platform provides a method for sustained expression of the CRISPR/Cas9 components, these results highlight the importance of identifying potent combinations of sgRNAs to induce activation in diverse cell types.

## DISCUSSION

CRISPR/Cas9 technologies are powerful tools for genomics, genetics and gene therapy. In this study, we adapted the Golden Gate cloning method to create a facile platform to rapidly generate single lentiviral Cas9 constructs for multiplex gene manipulation. This platform readily enabled multiplex gene editing and transient or sustained transcriptional activation in human cells. A single lentiviral CRISPR/Cas9 system is useful for both basic science and therapeutic applications by enabling highly efficient and tunable genomic manipulations in cell types that are difficult to transfect, such as primary cells and progenitor cells.

Importantly, this approach takes advantage of the simplicity and low cost of sgRNA design and should advance high-throughput genomic research using CRISPR/Cas9 technology (48–50) by targeting multiple loci with a single vector. Additionally, the vector will be useful for using multiplex gene editing for modelling complex diseases caused by combinations of mutations (51). For example, the single lentiviral vectors described here are useful in expressing Cas9 and numerous sgRNAs in diverse cell types, such as primary human dermal fibroblasts (Figure 6). In addition to transcriptional activation and nuclease functionality, this system will be useful for expressing other novel Cas9-based effectors that control epigenetic modifications for diverse purposes, including interrogation of genome architecture and pathways of endogenous gene regulation (21–24,35). As endogenous gene regulation is a delicate balance between multiple enzymes, multiplexing Cas9 systems with different functionalities will allow for examining the complex interplay among different regulatory signals. The vector described here is compatible with aptamer-modified sgRNAs (34) and orthogonal Cas9s (52,53) to enable increased complexity of genome engineering operations.

The system presented here effectively modifies or activates target genes with relatively high efficiency. However, some applications may require higher levels of gene modification or gene activation. To achieve higher multiplicity of infection and thus higher levels of transgene expression, the multiplex lentivirus could be concentrated using methods such as ultracentrifugation, or transduced cells could be sorted for high levels of GFP expression (44). Given the large packaging capacity of lentiviral vectors, this system could be expanded to include additional sgRNAs to increase the potency or tunability of this system. Furthermore, engineering enhanced minimal polymerase III-based promoters may enable more efficient expression of sgRNAs and expression of additional sgRNAs by reducing the size requirements for expressing individual sgRNAs. Since multiplex genome editing applications only require transient Cas9 expression to induce targeted genetic modifications, it would be advantageous to control the duration of Cas9 nuclease expression to decrease the possibility of unwanted off-target modifications. To address these concerns, Cas9 can be expressed under the control of a chemically inducible promoter (49). Similarly, Cas9-based transcriptional activators can be combined with methods for achieving inducible control by chemical (54,55) or optogenetic (22,56) regulation, to facilitate investigation of the dynamics of gene regulation in both time and space. Moreover, this system can be adapted for the production of integrase-deficient lentivirus (IDLV), which remains episomal and therefore results in transient expression of the lentiviral gene cassette. IDLVs are a viable alternative to achieve high levels of transduction in difficult-to-transfect and non-dividing cell types and can be used to transiently deliver nucleases in human cells (57).

Recent studies have described alternative methods for expressing multiple sgRNAs based on RNA processing, including the use of site-specific RNA endonucleases, ribozymes and introns (58–60). The type III CRISPR/Cas-associated Csy4 protein from *Pseudomonas aeruginosa* cleaves RNA following a 28-nt sequence. By including this sequence upstream of the sgRNA, a single transcript can be processed into multiple sgRNAs and also expressed from a Pol II promoter (58,59). However, a limitation of this approach is that the Csy4 RNA endonuclease must also be expressed. Additional studies are necessary to examine adverse effects of Csy4 expression on mammalian cells, although preliminary studies have already observed cell death at high expression levels (59). Ribozymes have also been used to process sgRNAs expressed from Pol II promoters (59,60), but activity is significantly lower than when expressed from Pol III promoters, presumably due to inefficient ribozyme cleavage or lower expression levels. Nevertheless, these systems benefit from the ability to use Pol II promoters, such that sgRNAs can be co-expressed with transgenes, including Cas9 variants, from a single transcript and well-characterized inducible promoters can be used for engineering gene circuits (59). Therefore each of these systems, including the single multiplex vector with multiple Pol III promoters described here, has unique strengths and weaknesses that will need to be considered for each application.

Advances in lentiviral technologies have abrogated many of the safety concerns associated with early retroviral delivery systems. Early viral therapies utilized retroviral vectors with intact long terminal repeats (LTRs) containing strong promoters that could interfere with endogenous genes (61). Furthermore, retroviral vectors preferentially integrate into promoter and enhancer regions, causing an increased incidence of oncogenesis by interfering with endogenous regulatory pathways (61). More recently, lentiviral vectors that exhibit less genotoxicity compared to retrovirus have been widely adopted (61). Furthermore, HIV-1-based lentiviral vectors integrate randomly into active transcription units, rather than preferentially into regulatory regions. This decreases the incidence of aberrant gene expression profiles (61). Newer lentiviral vectors contain self-inactivating LTRs to further increase their safety (61). These advances, including numerous preclinical and clinical successes of lentiviral gene therapy, have made lentiviral vectors a standard gene delivery vehicle for diverse applications.

The advent of engineered nucleases and transactivators has facilitated genome manipulation for the fields of gene therapy, synthetic biology, genomics and genetics. The CRISPR/Cas9 system has greatly simplified the engineering of novel DNA-binding enzymes and enabled low-cost access of these tools to the general biomedical research community. Applying these tools to reverse engineering of the genome will be highly valuable for understanding the complexity of gene regulatory pathways. Continued development of these technologies is necessary to meet the needs of the next era of high-throughput biomedical research. The facile single CRISPR/Cas9 expression system described here will be useful for reaching these goals by enabling rapid, tunable and efficient manipulation of genetic sequences and transcriptional activation.

## SUPPLEMENTARY DATA

Supplementary Data are available at NAR Online.

SUPPLEMENTARY DATA
