# Supplementary material for: Multiplex CRISPR/Cas9-based genome engineering from a single lentiviral vector
Source: Nucleic Acids Res. 2014 Aug 13;42(19):e147. doi: 10.1093/nar/gku749 (PMC4231726; doi:10.1093/nar/gku749)
Supplement: SUPPLEMENTARY DATA [file supp_42_19_e147__index.html]

Multiplex CRISPR/Cas9-based genome engineering from a single lentiviral vector — SUPPLEMENTARY DATA 

# Multiplex CRISPR/Cas9-based genome engineering from a single lentiviral vector

## SUPPLEMENTARY DATA

**Files in this Data Supplement:**

- SUPPLEMENTARY DATA
