# Supplementary material for: Multiplex CRISPR/Cas9-based genome engineering from a single lentiviral vector
Source: Nucleic Acids Res. 2014 Aug 13;42(19):e147. doi: 10.1093/nar/gku749 (PMC4231726; doi:10.1093/nar/gku749)
Supplement: SUPPLEMENTARY DATA [file supp_gku749_nar-01349-met-h-2014-File008.pdf]

Supplementary Information

Multiplex CRISPR/Cas9-Based Genome Engineering from a Single Lentiviral Vector

Ami M. Kabadi[♯1], David G. Ousterout[♯1], Isaac B. Hilton[1], and Charles A. Gersbach[1,2,3*]

[1]Department of Biomedical Engineering, Duke University, Durham, North Carolina, United States of America, 27708

[2]Center for Genomic and Computational Biology, Duke University, Durham, North Carolina, United States of America, 27708

[3]Department of Orthopaedic Surgery, Duke University Medical Center, Durham, North Carolina, United States of America, 27710

# The authors wish it to be known that, in their opinion, the first two authors should be regarded as joint First Authors.

* To whom correspondence should be addressed: Charles A. Gersbach Tel: 1-919-613-2147; Fax: 1-919-668-0795; Email: charles.gersbach@duke.edu

Present Address: [Charles A. Gersbach], Biomedical Engineering, Duke University, Durham, NC, 27708, USA

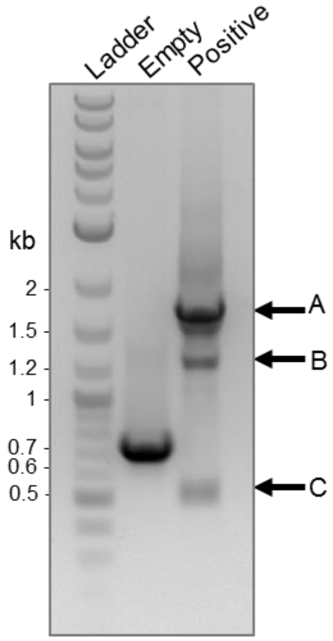

Supplementary Figure 1. Representative test PCR to screen for sgRNA-positive colonies. Empty vector and a final lentiviral vector containing four sgRNAs were amplified by PCR across the Golden Gate destination cassette using sense primer 5'-TCGGGTTTATTACAGGGACAGCAG-3' and antisense primer 5'-TCTAAGGCCGAGTCTTATGAGCAG-3'. Empty vector template should produce a PCR amplicon of approximately 700 base pairs in size. Due to the repetitive nature of the sgRNAs, a distinct banding pattern is observed in lentiviral vectors containing the four sgRNAs with the largest product approximately 1800 base pairs in size.

IL1RN

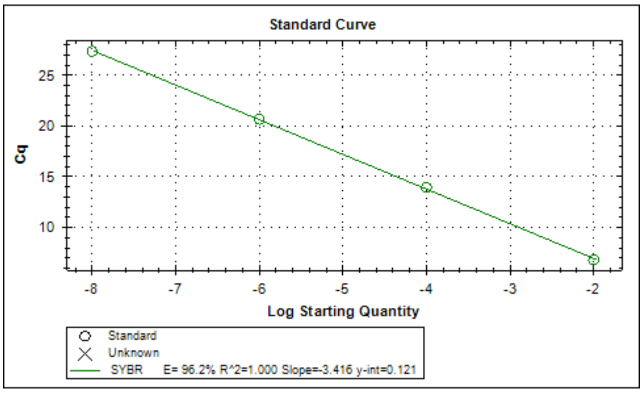

HBG1

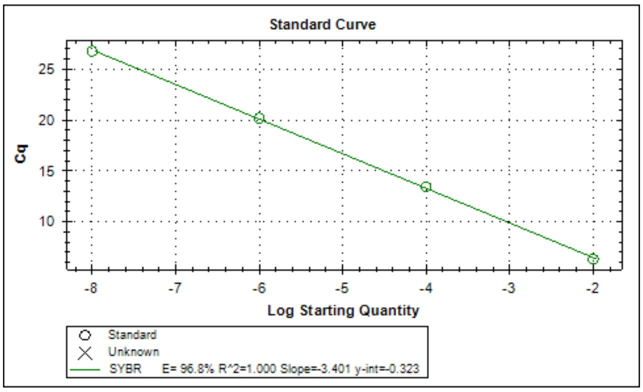

GAPDH

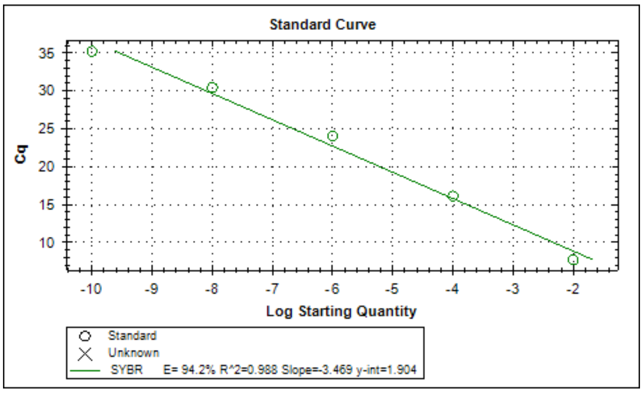

Supplementary Figure 2. qRT-PCR standard curves. To validate primers for each gene of interest, a positive and negative sample were analyzed by qRT-PCR to ensure linear amplification and product purity by melt curves. The positive and negative PCR products were run on an agarose gel to confirm that a single PCR band was detected at the expected size. The PCR band was gel purified and diluted to create a standard curve that was assayed by qRT-PCR to ensure efficient amplification over an appropriate dynamic range. The efficiencies of all amplification reactions were within 90-110%.

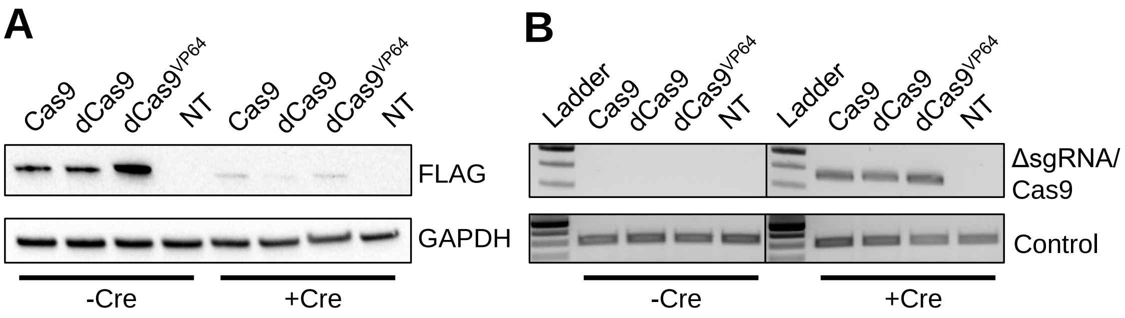

Supplementary Figure 3. Loss of Cas9 expression after Cre/loxP-mediated excision. The indicated constructs were delivered to HEK293T cells by lentiviral transduction. After 6 days, the lentivirus-treated cells were split for expression analysis (-Cre) or transfection with a Cre recombinase expression cassette (+Cre). Cre-treated cells were then collected 6 days after transfection (12 days total from initial transduction). (**A**) Western blot to detect expression of FLAG-tagged proteins for each indicated construct. (**B**) Genomic PCR to detect Cre-mediated excision of sgRNA and Cas9 expression cassettes integrated by lentiviral delivery (ΔsgRNA/Cas9). The loss of lentiviral sgRNA/Cas9 expression cassettes by Cre excision results in a 373 bp amplicon. Amplification of the AAVS1 locus was used as a positive control for the PCR reaction.

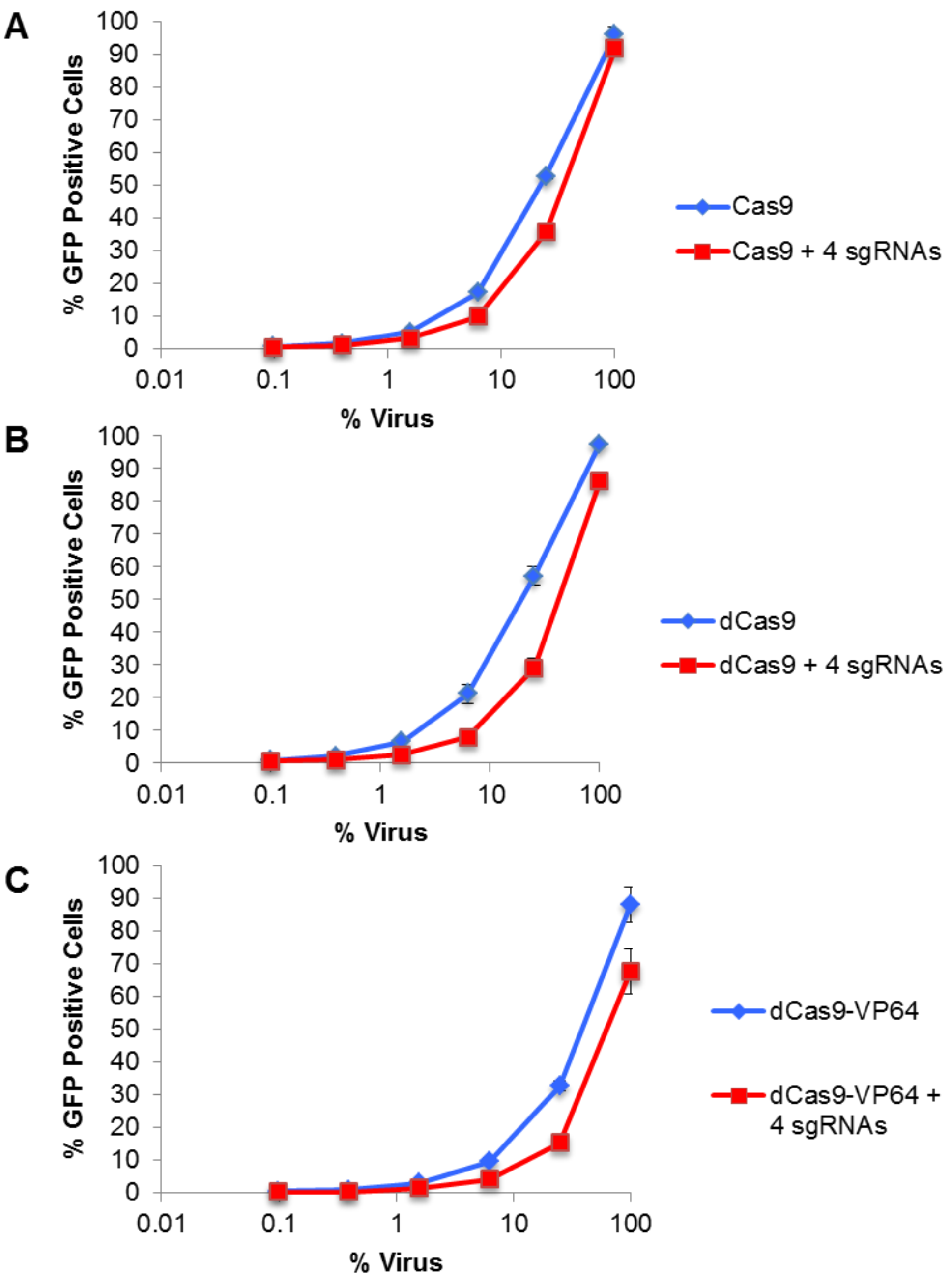

D

Condition	% Virus	% GFP Positive Cells	Titer (HEK293T transducing units/ml)
Active Cas9	1.56	5.20	1.66E+05
Active Cas9 + 4 sgRNAs	6.25	10.15	8.12E+04
dCas9	1.56	6.65	2.13E+05
dCas9 + 4 sgRNAs	6.25	7.95	6.36E+04
dCas9-VP64	6.25	9.75	7.80E+04
dCas9-VP64 + 4 sgRNAs	6.25	4.35	3.48E+04

Supplementary Figure 4. Titer of lentiviral vectors. HEK293T cells were transduced with each of the indicated lentiviral vectors at varying concentrations, and titer was determined as described in the Methods.

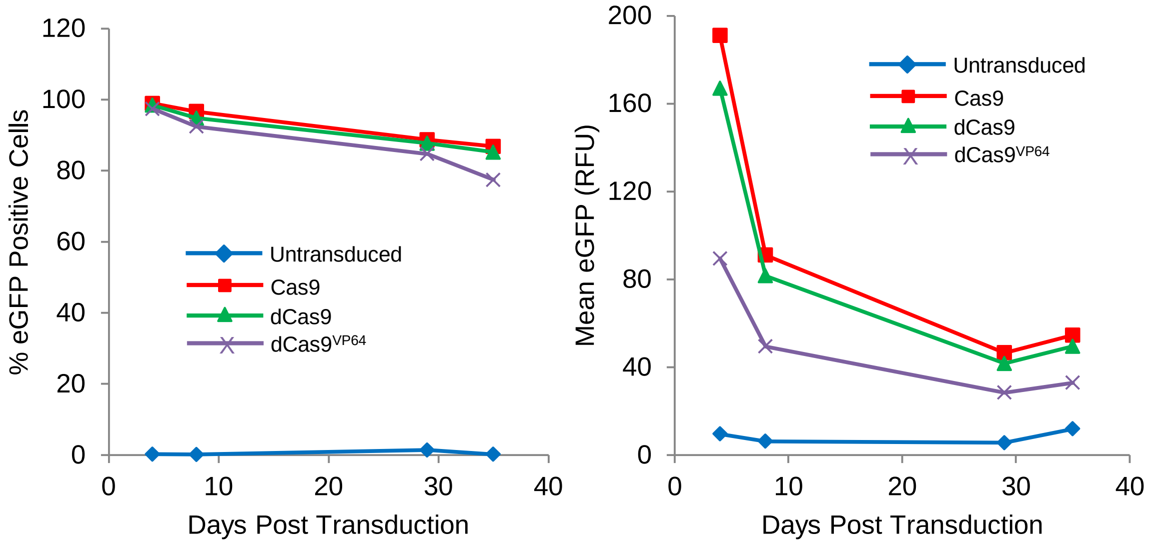

Supplementary Figure 5. Stable Cas9-T2A-GFP expression. HEK293T cells were transduced with different Cas9-T2A-GFP lentiviral vectors and eGFP expression was monitored using flow cytometry. Following normal passaging every 2-3 days, each cell line exhibited stable eGFP expression for up to 35 days post-transduction.

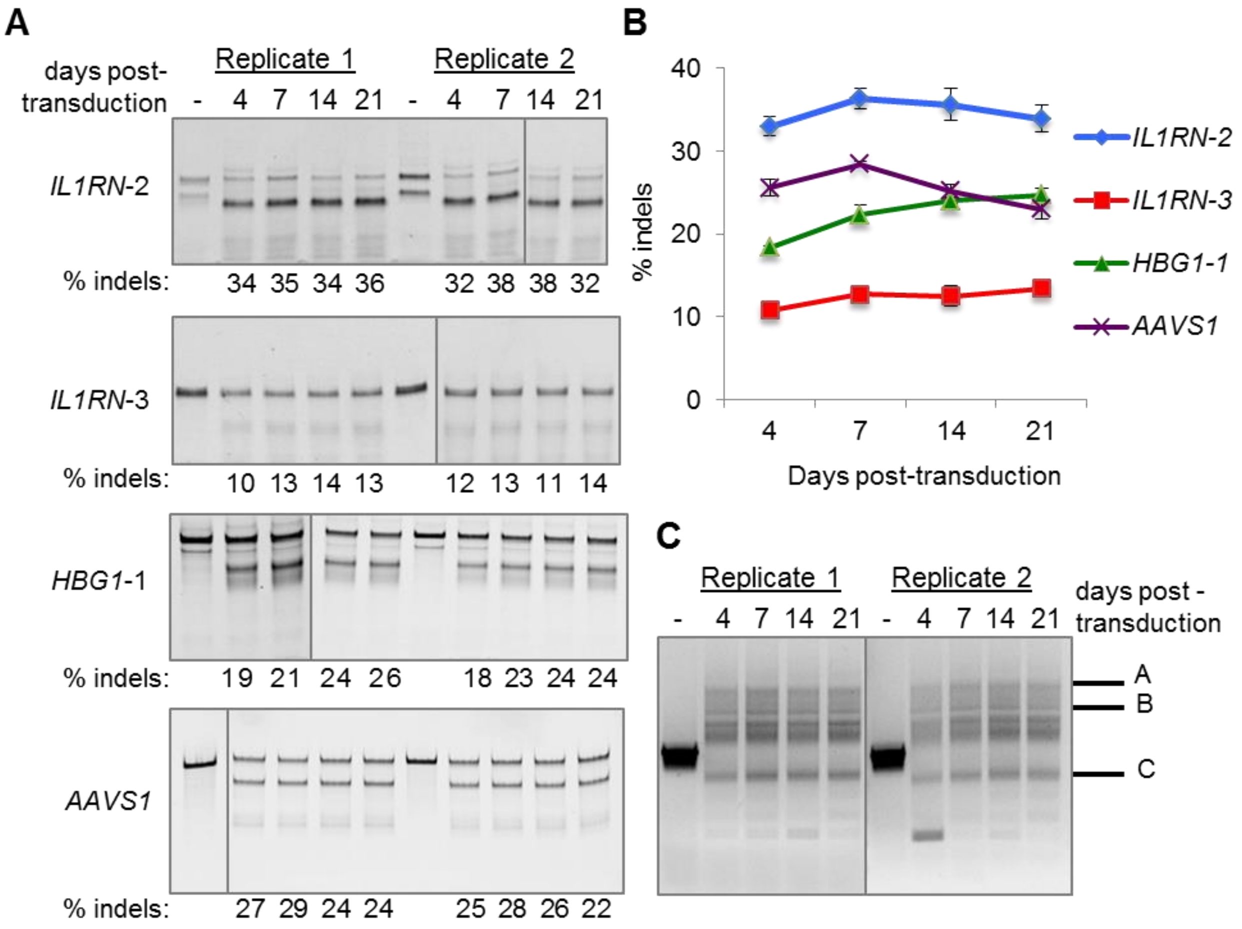

Supplementary Figure 6. Stable genome editing activity in HEK293T cells following transduction with lentivirus expressing active Cas9 nuclease and 4 sgRNAs. (A) Surveyor assay in a bulk population of HEK293T cells transduced with a single lentivirus to express Cas9 and 4 sgRNAs. Gene modification rates were assessed at 4, 7, 14, and 21 days after transduction and compared to cells treated with lentivirus expressing Cas9 only (-). (B) Plot of data from two replicates in (A) showing gene editing rates across 21 days following transduction with lentivirus expressing Cas9 and 4 sgRNAs. Means and standard error of the mean (n=2) were calculated by combining the replicates in (A). (C) Genomic DNA was isolated from cells that were transduced with a single lentivirus encoding active Cas9 nuclease and 4 sgRNAs. The DNA was amplified by PCR as in Supplementary Figures 1 and 7, with letters indicating the approximate size of the bands detected in those figures.

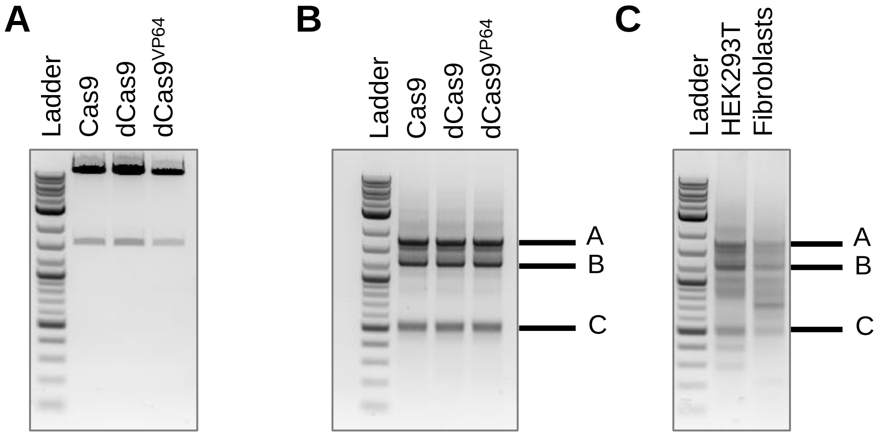

Supplementary Figure 7. Verification of integrity of sgRNA expression cassettes in plasmids used to generate lentivirus and in genomic DNA from cells transduced with lentivirus. (A) Lentiviral transfer plasmids for the indicated Cas9 constructs containing 4 sgRNA expression cassettes were digested with PacI restriction enzyme. Two PacI sites are present in the plasmids and flank the sgRNA expression cassettes. The presence of only the single band containing all four sgRNA expression cassettes, along with the plasmid backbone, indicates the plasmids are relatively pure with undetectable levels of recombination between sgRNA sequences. (B) Each lentiviral transfer plasmid from (A) was also amplified by PCR across the region containing all of the sgRNA cassettes, producing a pattern similar to the PCR results in **Supplementary Figure 1.** Because the restriction digest in (A) showed that the plasmids were intact, the PCR bands in (B) likely represent a characteristic pattern due to strand switching between similar sgRNA sequences during PCR. (C) Genomic DNA isolated from cells transduced with active Cas9 nuclease and 4 sgRNA cassettes from Figure 3A and PCR amplified as in (B), with bands of similar size as detected in (B).

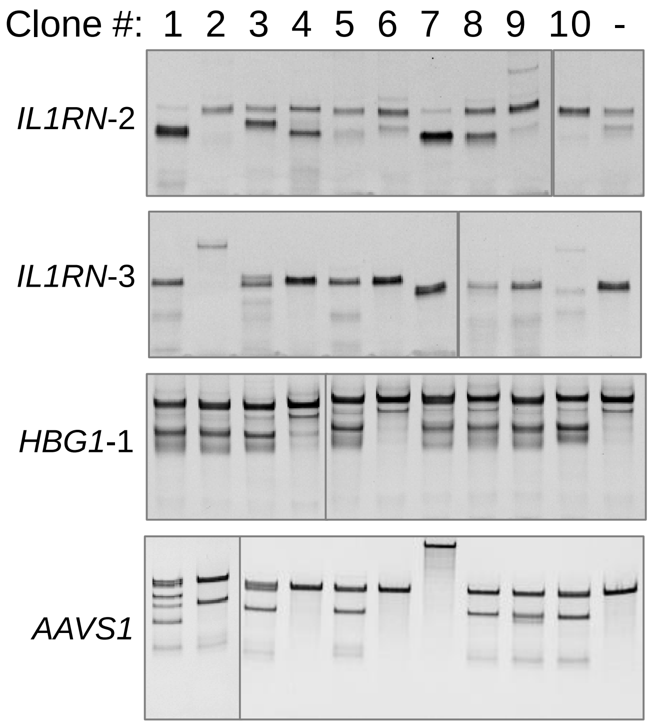

Supplementary Figure 8. Multiplex gene editing in single cells. Characterization of multiplex gene editing at four distinct loci in clonally derived HEK293T cell populations transduced with a single lentivirus carrying Cas9 and 4 sgRNA expression cassettes as in Figure 3A. Genomic DNA from 10 different clones was isolated and gene editing activity at all four loci was assessed using the Surveyor assay. A table summarizing detected on-target activity at each locus across all clones is presented in Figure 3C. Note that distinct modifications may be occurring at different alleles and that deletions can potentially occur following concurrent cleavage at *IL1RN*-2 and *IL1RN*-3 target sites.

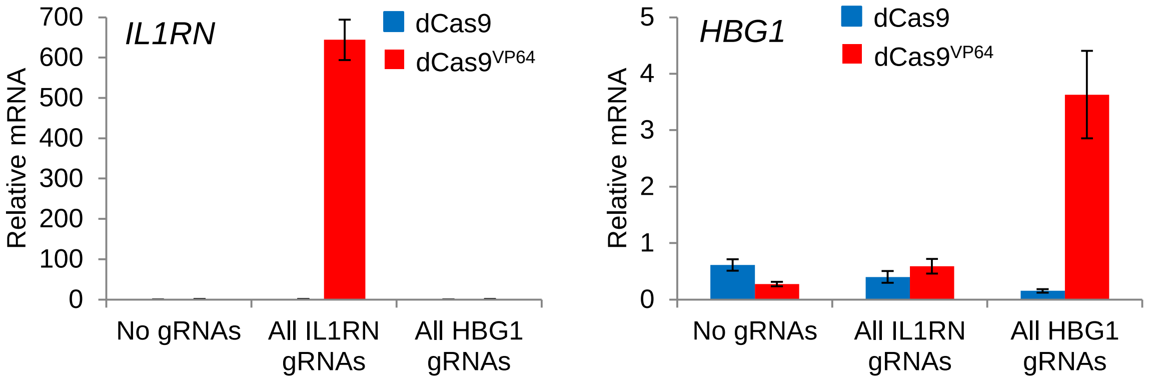

Supplementary Figure 9. sgRNAs are specific for desired target. HEK293T cells were transduced with lentivirus to stably express dCas9-T2A-GFP or dCas9^{VP64}-T2A-GFP and were subsequently transfected with plasmids expressing the indicated sgRNA combinations. Three days post-transfection, cells were harvested for qRT-PCR analysis. The *HBG1* and *IL1RN* sgRNAs only activate their respective targets.

Promoter construct	Oligo	Overhang	Protospacer	Overhang
mU6	sgRNA-1 sense	5'-TTGTTTG	(N)20	
	sgRNA-1 antisense	3'-AAC	(N)20 complement	CAAA-5'
hU6	sgRNA-2 sense	5'-CACCG	(N)20	
	sgRNA-2 antisense	3'-C	(N)20 complement	CAAA-5'
7SK	sgRNA-3 sense	5'-CCTCG	(N)20	
	sgRNA-3 antisense	3'-C	(N)20 complement	CAAA-5'
H1	sgRNA-4 sense	5-'TCCCA	(N)20	
	sgRNA-4 antisense	3'-T	(N)20 complement	CAAA-5'

Supplementary Table 1. Design of oligonucleotides for cloning new protospacers into sgRNA expression cassettes.

Target	Forward Primer	Reverse Primer	Use	PCR Conditions	
AAVSI sgRNA transcript	GGGGCCACTAGGGACAGGAT	CCGACTCGGTGCCACTTTTT	Conventional RT-PCR	95°C 5 min<br>95°C 30 sec<br>55°C 30 sec<br>68°C 30 sec<br>68°C 5 min	35X
IL1RN-1 sgRNA transcript	CCTCGTGTACTCTCTGAGGTGCTC	CCGACTCGGTGCCACTTTTT	Conventional RT-PCR	95°C 5 min<br>95°C 30 sec<br>55°C 30 sec<br>68°C 30 sec<br>68°C 5 min	35X
IL1RN-2 sgRNA transcript	CACCGACGCAGATAAGAACCAGTT	CCGACTCGGTGCCACTTTTT	Conventional RT-PCR	95°C 5 min<br>95°C 30 sec<br>55°C 30 sec<br>68°C 30 sec<br>68°C 5 min	35X
IL1RN-3 sgRNA transcript	TTGTTTGCATCAAGTCAGCCATCAGC	CCGACTCGGTGCCACTTTTT	Conventional RT-PCR	95°C 5 min<br>95°C 30 sec<br>55°C 30 sec<br>68°C 30 sec<br>68°C 5 min	35X
IL1RN-4 sgRNA transcript	TCCCAGAGTCACCCTCCTGGAAAC	CCGACTCGGTGCCACTTTTT	Conventional RT-PCR	95°C 5 min<br>95°C 30 sec<br>55°C 30 sec<br>68°C 30 sec<br>68°C 5 min	35X
GAPDH	CAATGACCCCTTCATTGACC	TTGATTTTGGAGGGATCTCG	Conventional RT-PCR	95°C 5 min<br>95°C 30 sec<br>55°C 30 sec<br>68°C 30 sec<br>68°C 5 min	35X
IL1RN	GGAATCCATGGAGGGAAGAT	TGTTCTCGCTCAGGTCAGTG	qRT-PCR	95°C 30 sec<br>95°C 5 sec<br>58°C 20 sec	45X
HBG1	GCTGAGTGAACTGCACTGTGA	GAATTCTTTGCCGAAATGGA	qRT-PCR	95°C 30 sec<br>95°C 5 sec<br>58°C 20 sec	45X
GAPDH	CAATGACCCCTTCATTGACC	TTGATTTTGGAGGGATCTCG	qRT-PCR	95°C 30 sec<br>95°C 5 sec<br>53°C 20 sec	45X
HBG1	CCTGGCTAAACTCCACCCAT	CAGACGCCATGGGTCATTTC	Surveyor	94°C 5 min<br>94°C 30 sec<br>56°C 30 sec<br>68°C 40 sec	35X
IL1RN-2	AAGGCCCAGCTCAGTTCTCT	CTGAGTCACCCTCCTGGAAA	Surveyor		
IL1RN-3	GAAACTGGGCCTGCTTGG	CCTCTGTGAGTGTGTGGGAG	Surveyor		
AAVS1	CATGGCATCTTCCAGGGGT	GGAATCTGCCTAACAGGAGGTG	Surveyor		
sgRNA PCR	TCGGGTTTATTACAGGGACAGCAG	TCTAAGGCCGAGTCTTATGAGCAG	PCR across gRNA region	95°C 5 min<br>95°C 30 sec<br>56°C 30 sec<br>68°C 2 min<br>68°C 5 min	35X

Supplementary Table 2. PCR primers and conditions.
